# The Role of Biodegradable Temporizing Matrix in Paediatric Reconstructive Surgery

**DOI:** 10.3390/jcm14155427

**Published:** 2025-08-01

**Authors:** Aikaterini Bini, Michael Ndukwe, Christina Lipede, Ramesh Vidyadharan, Yvonne Wilson, Andrea Jester

**Affiliations:** 1 Hand and Upper Limb Service, Department of Paediatric Plastic, Hand & Reconstructive Surgery, Birmingham Women’s and Children’s NHS Foundation Trust, Birmingham B4 6NH, UK; mndukwe1@sheffield.ac.uk (M.N.); christina.lipede2@nhs.net (C.L.); rvidyadharan@nhs.net (R.V.); andrea.jester@nhs.net (A.J.); 2 Burns Centre, Birmingham Women’s and Children’s NHS Foundation Trust, Birmingham B4 6NH, UK; yvonne.wilson26@nhs.net

**Keywords:** biodegradable temporizing matrix, BTM, skin substitute, dermal substitute, reconstructive surgery, paediatric patients

## Abstract

**Introduction:** Biodegradable Temporizing Matrix (BTM) is a new synthetic dermal substitute suitable for wound closure and tissue regeneration. The data in paediatric population remain limited. The study purpose is to review the indications for BTM application in paediatric patients, evaluate the short-term and long-term results, including complications and functional outcomes, as well as to share some unique observations regarding the use of BTM in paediatric population. **Patients and Methods:** Patients undergoing reconstructive surgery and BTM application during the last three years were included. Data collected included patient demographics, primary diagnosis, previous surgical management, post-operative complications and final outcomes. BTM was used in 32 patients. The indications varied including epidermolysis bullosa (n = 6), burns (n = 4), trauma (n = 7), infection (n = 4), ischemia or necrosis (n = 11). **Results:** The results were satisfying with acceptable aesthetic and functional outcomes. Complications included haematoma underneath the BTM leading to BTM removal and re-application (n = 1), BTM infection (n = 1) and split-thickness skin graft failure on top of BTM requiring re-grafting (n = 2). **Conclusions:** BTM can be a good alternative to large skin grafts, locoregional flaps or even free flaps. The big advantages over other dermal substitutes or skin grafts are that BTM is less prone to infection and offers excellent scarring by preserving the normal skin architecture. Specifically in children, BTM might not require grafting, resulting in spontaneous healing with good scarring. In critically ill patients, BTM reduces the operation time and there is no donor site morbidity. BTM should be considered in the reconstructive ladder when discussing defect coverage options in children and young people.

## 1. Introduction

Biodegradable Temporizing Matrix (BTM) has been proven to be a promising development in the treatment of complex defects. It is a versatile tool, with application in extremity avulsion injuries, burns, necrotising fasciitis, tumour excision, open fractures, ulcers and chronic wounds [1,2,3,4,5,6]. However, there is a lack of current literature regarding its application in paediatric patients.

The treatment of complex wounds has posed a consistent challenge, especially in children [7]. Obtaining a balance between functional and aesthetic outcomes while avoiding complications is vital [7]. Previously established surgical methods for dealing with wound defects include flaps, split-thickness skin grafts (STSG), full-thickness skin grafts (FTSG) and dermal substitutes, especially in cases where skin grafts alone cannot cover the defect [2,3]. There are multiple skin substitutes available, which can be classified by their composition, from animal-derived or human-derived to completely synthetic products [3]. Integra is a synthetic substitute widely known in the management of paediatric complex wounds; however, its use is limited due to shrinkage and infection rates [2].

Novosorb BTM is a 2 mm thick, completely synthetic skin substitute composed of two main components [8]. The first one is a biodegradable polyurethane foam acting as a scaffold to allow vascular ingrowth and cell proliferation [3]. Once the neodermis is established, this foam gradually dissolves through hydrolysis [4,9]. The second component is a transparent non-biodegradable polyurethane sealing membrane containing small fenestrations [3,8]. The sealing membrane provides a temporary physical and physiological wound barrier [3]. This limits moisture loss via evaporation and reduces wound contraction, while allowing drainage through the fenestrations [3,7,8].

The application of BTM is usually carried out in a two-stage process [7]. In the first stage, BTM is applied to the clean, debrided wound bed [3,7]. Once the foam matrix is integrated, the sealing membrane is removed [3,7,8]. An STSG is most often used in the second stage to cover the wound [7]. The interval between the initial BTM application and the STSG application ranges from 3 to 5 weeks depending on the integration speed [3,7,10].

The current literature points to BTM application in a wide range of scenarios with a low complication rate [7,8,9]. Infection in BTM use seems less common than this with other dermal substitutes and its synthetic composition results in bacterial colonisation still being compatible with good outcomes [7,8].

In paediatric patients, BTM has been applied in a range of anatomical areas, most commonly on the upper and lower limbs [6,9]. The functional results are also promising, with patients being able to return to previous levels of activities with good mobility [3,4]. Despite its potentials, there is still a lack of clarity on the optimal BTM indications and applications, as well as its differences in comparison to adult patients [9].

The study purpose is to review the indications of BTM application in paediatric patients, evaluate the short-term and long-term results, including complications and functional outcomes, as well as to point out the unique observations in children, including cases requiring no grafting due to healing process close to normal texture, conditions not intended to be grafted (e.g., epidermolysis bullosa) and differences to the adult population.

## 2. Patients and Methods

The present study is a retrospective study. This study was registered with the Institution’s Research & Development office and in accordance with the UK National Research Ethics Service guidance, neither individual informed consent nor formal research ethics committee review was required as the study was undertaken by the direct clinical care team using information previously collected in the course of routine care (Local Reference: 37/BWC/LA/Bini). Details regarding personal information and identification remain anonymous and confidential. No recognizable features are included in the illustrations. Written consent was obtained from all the patients for the publication of their illustrations.

The selection criteria included all paediatric patients who underwent surgery and BTM application within the last three years (2022–2025). The indications varied including epidermolysis bullosa (n = 6), burns (n = 4), trauma (n = 7), infection (n = 4), ischemia/necrosis (n = 11).

There were five cases of recessive dystrophic epidermolysis bullosa (RDEB) undergoing hand surgery for webspaces and flexion contracture release and an RDEB case affecting the right external ear. Regarding burn injuries, two cases of scald burn, a case of flame burn and a case of contact burn were included. Trauma cases included three avulsion/degloving injuries, two friction burns, a crush injury and a dog bite injury. There were also four cases requiring BTM application after tissue infection: a case of great toe osteomyelitis due to biting, a case of multifocal osteomyelitis due to staphylococcal infection, a case of infected pilomatrixoma and a case of infection of unknown origin.

The most common aetiology for BTM use was defect reconstruction after tissue ischemia or necrosis due to streptococcal A septicaemia (n = 4), meningococcal septicaemia (n = 1), necrotising fasciitis after chickenpox (n = 1), compartment syndrome after extravasation injury (n = 2), neonatal skin ischemia (n = 1) and neonatal compartment syndrome (n = 2). Neonatal ischemia/compartment syndrome occurred in two pre-term neonates below the age of 30 weeks who suffered from gangrene of the extremities, where BTM was used to cover the amputated stumps.

The anatomical site also varied including hand and upper limb in the majority of cases (n = 20), followed by lower limb (n = 12), craniofacial area (n = 2) and trunk (n = 1).

The surgical technique included wound preparation, debridement, then application of equal piece of BTM and securing with vicryl rapide in the majority of cases. Non-absorbable sutures like prolene (n = 2), ethilon (n = 1) and clips (n = 1) were rarely used. BTM was mainly dressed with silver dressing to minimise infection risk. Negative pressure wound therapy (NPWT) was applied in 14 cases. The decision for NPWT application depended on the anatomical site involved including mainly amputated stumps (Patient 22, Patient 23 and Patient 28), challenging anatomical areas which are not flat and might delay BTM incorporation (e.g., ankle in Patient 7, Patient 8 and Patient 20; dorsum of foot in Patient 8 and Patient 27; elbow in Patient 17; wrist in Patient 32) and large wounds (thigh in Patient 11; lower leg in Patient 14; forearm in Patient 21 and Patient 26; upper back in Patient 24).

The time interval between BTM application and delamination followed by grafting varied between 23 and 42 days, depending on BTM incorporation, granulation tissue formation, the defect size and location as well as the patient’s clinical status. BTM incorporation or integration refers to the cellular migration which enables new blood vessel formation and collagen production throughout the matrix. Integration time varies depending on patient and wound factors.

There were 16 cases requiring no grafting, including RDEB (n = 6), dog bite injury (n = 1), hand trauma (n = 2), osteomyelitis (n = 1), soft tissue infection (n = 1), compartment syndrome (n = 2), amputation (n = 1), congenital ischemia (n = 1) and skin necrosis (n = 1). Several parameters contributed to the decision not to graft including the condition itself (e.g., RDEB in Patient 1, Patient 2, Patient 3, Patient 4, Patient 5 and Patient 6), the age of the patient (e.g., pre-term neonates—Patient 19, Patient 29, Patient 30 and Patient 31), the size of the defect and its reduction by using NPWT (Patient 11, Patient 26 and Patient 32), the anatomical area (e.g., dorsum of hand—Patient 13; finger—Patient 16; toe—Patient 18) and the mechanism of injury (e.g., friction burn in Patient 13).

The study evaluates the short-term as well as the long-term results. The short-term results are evaluated within the first two months, during the perioperative period, focusing on the efficient BTM incorporation and skin grafting, including post-operative complications. The long-term results are evaluated within two years post-operatively, focusing mainly on the functional outcome, the necessity for occupational therapy and splinting, as well as the aesthetic appearance of the scar. The assessment methods regarding functional and aesthetic outcomes depended on how these affected the daily activities of the patients (function) as well as the aesthetic perception and self-esteem (cosmesis), (Table 1).

## 3. Results

During the study interval (2022–2025) the medical records of 32 consecutive patients who underwent reconstructive surgery with BTM application were reviewed. Demographic data were recorded. There were 16 males and 16 females with an age range from 2 days old to 16 years old (median age: 6.4) when undergoing operation with BTM application (Table 1).

Regarding the four RDEB patients who underwent hand surgery for finger contractures and webspace release, the final outcome depended on the compliance with post-operative occupational therapy (OT) and splinting. As RDEB inevitably causes finger refusion and contractures, BTM application aims to maintain as long as possible the thumb abduction, release the second-to-forth webspaces and fused fingers and in this way prolongs the time interval between surgery and recurrence. Three patients with bilateral hand release did not initially comply with OT and splinting, requiring re-operation (Patient 2, Patient 3 and Patient 4). After re-operation better functional results were noticed due to compliance with OT and splinting. BTM was also used in an RDEB patient after a biopsy of suspected squamous-cell carcinoma in the right middle fingertip, with good healing process and no malignancy in the histology report (Patient 5). There was also a patient with RDEB affecting the right ear, where BTM was used to cover the exposed cartilage (Patient 6). However, satisfying healing process was not achieved due to chronic, recurrent wounds caused by the condition itself. Second stage of grafting was not performed in any of the RDEB patients, as skin grafts are not only blistered and fragile and therefore unsuitable, but also the donor site does not heal properly.

Regarding burn patients, there were two scalds, one flame and one contact burn. The anatomical areas included the right ankle (n = 1), the left ankle and the dorsum of the right foot (n = 1), the right palm (n = 1) and the forearms bilaterally (n = 1). Indications for BTM application were post-burn contracture release in the two cases of lower leg involvement, failure of FTSG in the case of right palm contact burn and critical condition/unsuitability for grafting in the case of bilateral forearm involvement. Second stage of grafting was performed in all these cases, combined with NPWT in two cases involving the ankle joint and the dorsum of the foot. The patient with bilateral foot involvement developed tight scars in both anterior ankles restricting full active range of motion (ROM) and requiring triamcinolone acetonide (TCA) injections, ongoing OT and thermoplastic splint (Patient 8). The patient with the right palm burn developed severe finger contractures requiring ongoing OT and splinting (Patient 9). In the case of bilateral forearm involvement, BTM was complicated with infection, requiring removal 30 days after application (Patient 10).

**Figure 1:**
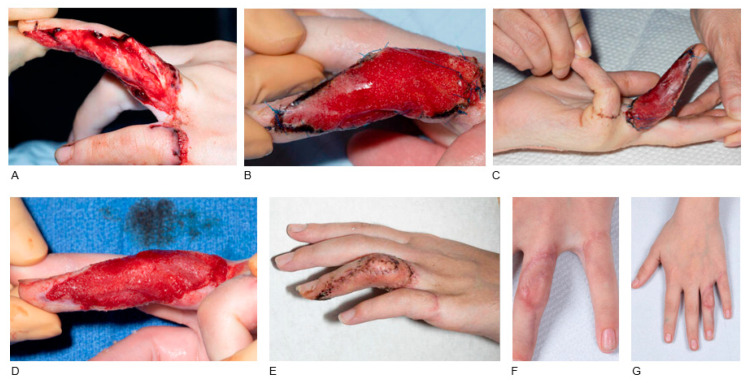
BTM application in avulsion injury of the left ring finger. A 12-year-old female patient who sustained an avulsion/degloving injury in the ulnar aspect of left ring finger. The ulnar digital nerve, which was repaired with an interposition vein graft, the extensor and flexor tendons were exposed (**A**). Defect reconstruction and structures coverage were achieved with BTM application. BTM was secured with 5.0 prolene (**B**,**C**). BTM was delaminated after 23 days. BTM incorporation and healthy granulation tissue were noticed (**D**). A full-thickness skin graft was applied. Post-operative appearance of the left ring finger 20 days post-grafting with fully taken graft and normal healing process (**E**). Clinical appearance three months post-operatively, with tight scar formation causing stiffness at proximal and distal interphalangeal joints and requiring ongoing occupational therapy and splinting (**F**,**G**).

**Figure 2:**
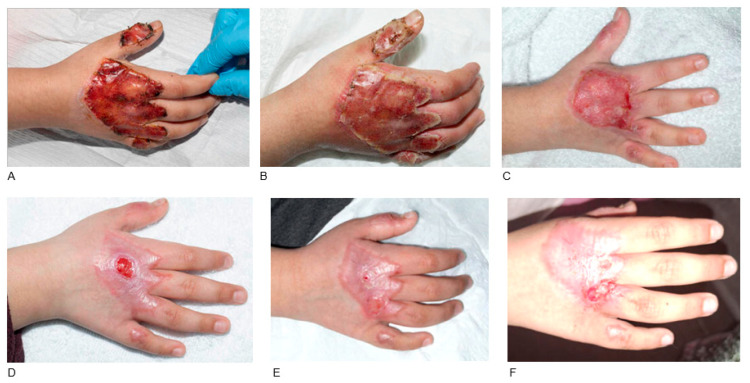
BTM application in friction burn in the dorsum of the right hand without grafting. A 6-year-old female patient who sustained a friction burn in the dorsum of the right hand over the MCP joints and thumb interphalangeal joint with exposed extensor tendons. BTM was applied (**A**). BTM integration was normal and BTM was ready for delamination 23 days post-operatively (**B**). Gradual healing process without grafting (**C**–**E**). Wound fully healed within three months after BTM application requiring occupational therapy for scar management (**F**).

**Figure 3:**
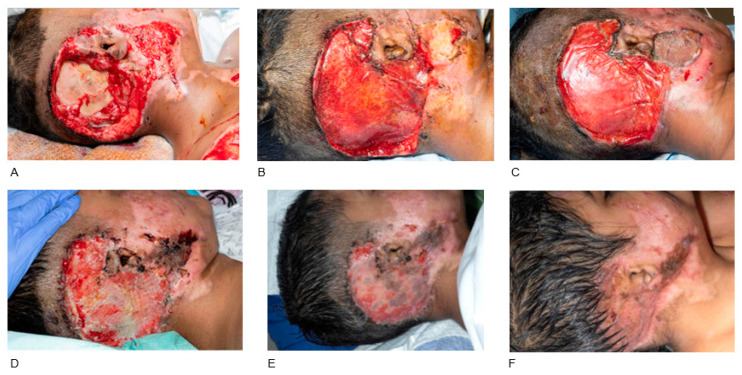
BTM application in a degloving injury of the right occipital bone. A 10-year-old female patient who sustained a road traffic accident, resulting in multiple degloving injuries. Clinical appearance of the wound in the right occipital bone on the date of injury, with exposed calvarium (**A**). BTM application for occipital bone coverage 15 days after the initial injury (**B**). BTM incorporation 14 days later (**C**). An STSG was applied 24 days post BTM application. Skin graft failure on top of BTM 12 days later, requiring re-grafting (**D**). Second STSG fully taken 12 days after re-grafting (**E**). The final result three months post-operatively with satisfying healing process and acceptable scarring, given the nature and severity of the injury (**F**). Right ear reconstruction will be planned in the future.

Seven trauma cases required BTM application including avulsion/degloving injury (n = 3), friction burn (n = 2), crush injury (n = 1) and dog bite injury (n = 1). A full-thickness skin graft was applied in one case (Patient 12, Figure 1), while no grafting was required in two cases of BTM application in the hand (Patient 13, Figure 2; Patient 16) and one case of BTM application with NPWT in the thigh (Patient 11). Regarding complications, tight scars and stiffness of distal interphalangeal (DIP) and proximal interphalangeal (PIP) joints developed after BTM and FTSG in the case of left ring finger avulsion injury, which was managed with ongoing OT and splinting (Patient 12, Figure 1). There was also a case of BTM application in the right occipital bone, where the STSG failed requiring re-grafting (Patient 15, Figure 3). Haematoma underneath the BTM developed in a case of right index finger crush injury, which was treated conservatively without BTM removal. In this case a hypertrophic scar developed requiring ongoing OT (Patient 16).

Soft tissue loss due to infection occurred in four patients, including two cases of osteomyelitis. In two of these cases involving the right forearm and right medial ankle and lower leg, BTM was followed by STSG and NPWT (Patient 20 and Patient 21), while the other patients with defects in the distal phalanx of the right great toe and the left elbow joint required no grafting (Patient 18 and Patient 19). Post-operative outcomes included sensation loss in the right great toe due to osteomyelitis (Patient 18), tight left wrist extension, scarring and complete loss of median, radial and ulnar nerve function/neurolysis (Patient 19), as well as stiffness in the right ankle joint due to osteomyelitis (Patient 20).

**Figure 4:**
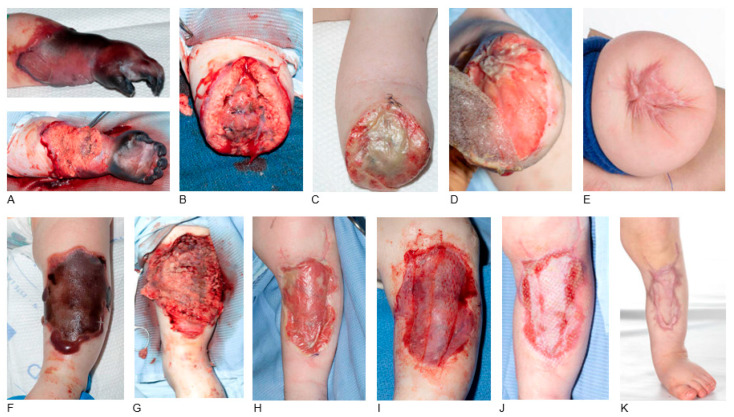
BTM application after left forearm amputation and left lower leg debridement. A 10-month-old male patient diagnosed with streptococcal A septicaemia and septic embolism to the left upper limb (**A**). Intra-operative image of left arm amputation below elbow with exposed bone (**B**). BTM was used for stump coverage. Eighteen days post BTM application with good incorporation (**C**). BTM delamination followed by STSG and NPWT 24 days post BTM application (**D**). The final outcome of the left upper limp stump five months post-operatively (**E**). Left lower leg anterior shin necrosis (**F**). Debridement down to the muscle layer and coverage with BTM (**G**). BTM incorporation 24 days post BTM application (**H**). A meshed STSG was applied after BTM delamination (**I**). Graft taken with small defects and overgranulation tissue in wound edges 25 days post-operatively (**J**). The final outcome five months post-operatively with scar formation without causing restriction in range of motion (**K**).

Critical conditions leading to multiorgan failure and peripheral ischemia/necrosis included four cases of streptococcal A septicaemia and a case of meningococcal septicaemia. Septic embolism caused tissue ischemia/necrosis resulting in limb amputation in three cases and extended debridement in all five cases (Patient 22, Figure 4). BTM, STSG and NPWT were used in the amputated stumps (n = 3), STSG without NPWT was applied in a defect in the anterior shin and the dorsum of the right foot, while no grafting was required in a case of full-thickness necrosis in the right distal forearm and wrist (Patient 32). Complications included haematoma underneath the BTM requiring removal and re-application (Patient 28).

Necrotising fasciitis after chickenpox occurred in one patient involving the upper back and was managed with BTM, followed by STSG and NPWT, with satisfying outcome (Patient 24). Compartment syndrome due to extravasation injury occurred in two cases in the right forearm; one was treated with BTM and NPWT without grafting (Patient 26), while the other one was a pre-term (28 weeks) neonate, which required below elbow amputation (bed-side) and BTM application in the stump without grafting (Patient 29). The second pre-term (24 weeks) neonate presented with congenital ischemia of bilateral feet, requiring bilateral forefoot amputation and BTM application in the stumps without grafting (Patient 31). Two patients developed neonatal compartment syndrome; one in the dorsum of the left foot and one in the right forearm, respectively. The first patient was a one-month-old boy who was treated with BTM, STSG and NPWT in the dorsum of the left foot. However, there was delayed graft failure 12 days post STSG and the histology revealed a non-involuting congenital haemangioma (Patient 27). The other case was a 9-day-old boy who underwent right forearm fasciotomies with BTM application without grafting. Sixteen months later contracture release with serial Z-plasty was performed along with neurolysis of the median nerve and tendon lengthening of the flexor carpi radialis (FCR) and flexor digitorum superficialis (FDS). The patient required casting for 2 years and developed scar contractures in the right wrist with intrinsic function of the ulnar nerve (Patient 30).

## 4. Discussion

This study evaluated the use of BTM in paediatric patients undergoing reconstructive surgery. The indications, surgical techniques, outcomes and complications were carefully analysed to provide a comprehensive understanding of the optimal management strategies.

BTM has shown promising results in a variety of clinical situations and wound types [6,7]. BTM use in paediatric patients is not limited by age; adolescents, toddlers and infants can be suitable candidates [2,5,7]. However, the existing literature rarely focuses solely on BTM application in children; instead, both paediatric and adult patients are included in the majority of studies.

BTM demonstrates versatility regarding the anatomical site, including both upper and lower extremities, head and neck, trunk and external genitalia [1,2,3,7,11]. BTM can be successfully applied on exposed bones, amputated stumps, tendons, muscles, fat, perichondrium, submandibular glands, carotid sheaths and periosteum [6]. BTM has also been used over joints, where skin grafts would lead to scarring and reduced ROM [1].

BTM can be placed on structures with little vascularisation and still integrate successfully [4,7]. However, BTM application over non-vascularised structures requires additional time for incorporation and neodermis formation. Poor vascularisation is one of the main contraindications for BTM use. Patients with peripheral vascular disease seem to be problematic and at higher risk [4]. That might be the case in adults, but potentially not in children.

Immunosuppressive therapy has also been highlighted as a statistically significant factor for poor BTM take [1]. Other relative contraindications include large joints, large blood vessels, the presence of synthetic material, radiated tissue and open fractures of large bones [1]. These mainly affect adults, but it is not proven in children as their whole physiology and pathophysiology are different.

BTM may be applied directly after debridement or at a later stage and may be secured in children with vicryl rapide rather than non-absorbable sutures [5]. In paediatric patients, the defect sizes suitable for BTM application vary, ranging from 10 cm^2^ to 480 cm^2^ [2].

BTM incorporation is tested by edging the silicon layer off; if it comes off, it is stipulated that it is ready for grafting. In smaller defects, much longer time should be allowed before delamination, as BTM might heal without skin grafting.

Negative pressure wound therapy (NPWT) has shown positive results, as it can speed up the granulation formation and aid incorporation [1,2,5,6,7]. Significantly more integration has been noticed in BTM with NPWT compared to BTM alone, as NPWT offers a better seal [12]. NPWT also leads to a decreased inflammatory response, creating a more favourable environment for BTM [12]. Nevertheless, NPWT has not been associated with a lower infection risk [6] and post-operative haemorrhage has been observed within the first 24 hours after NPWT use in large acute wounds that underwent BTM reconstruction [7].

Although BTM is less prone to infection in comparison to other dermal substitutes, bacterial colonization can occur reducing BTM survival [6,7]. However, one of the main advantages of BTM is its ability to still integrate despite the infection, without absolute need for removal [1]. Pseudomonas aeruginosa, Staphylococcus spp., Streptococcus pyogenes and Enterobacter spp. are the most common bacteria [6]. In case of infection, BTM can be gently lifted, washed out and then placed back, combined with antibiotics and close wound monitoring [1,4,7].

Haematoma is another complication that can lead to reduced BTM integration and skin graft failure [1,5,7,9]. Management of haematoma can vary. Options include making small incisions within the BTM to drain the haematoma [1], BTM removal and re-application [4,5] or BTM lifting to evacuate the haematoma without removal [1]. It is always important to restore contact between BTM and wound bed [5]. The present study has shown that BTM removal is not necessary in case of haematoma. In Patient 28, BTM was removed from the left thigh stump and left forearm due to haematoma, but was kept in the right upper arm, resulting in full take despite the haematoma underneath.

Overall published outcomes with BTM are generally positive with a mean percentage of BTM integration between 82.7% and 98% [1,5,6]. Re-operations after BTM application are usually rare, including BTM re-application or re-grafting with either STSG or FTSG [6,7]. Factors that may lead to re-grafting are wound infection and haematoma [7].

The current literature regarding BTM application in paediatric patients does not place much focus on aesthetic outcomes [3]. Nevertheless, good aesthetic outcomes and scarring results have been reported in several studies [1,2,7,9]. BTM can reduce wound contracture and improve scarring [6,13,14,15,16]. Tissue quality after BTM use has been described as “softer and more supple” compared to direct STSG [1]. The results of this study have proven satisfying aesthetic outcomes with acceptable scarring in the majority of children.

Alternative dermal substitutes are also available for complex wounds, which mainly include acellular dermal matrices (e.g., Integra, Matriderm, Nevelia, Biobrane). Integra, for example, is an established two-stage option consisting of purified collagen cross-linked with glycosaminoglycan [17]. Compared to other dermal substitutes, one frequently mentioned advantage of BTM is its robustness in case of infection, which is important as infection often leads to dermal substitute failure [4,7,18]. In addition to infection resistance, the completely synthetic composition of BTM means that there are fewer religious and cultural objections to its use [2]. BTM has been associated with reduced hospital stay, surgery time and revisionary surgery rates, as well as fewer follow-up visits compared to Integra [19]. These factors combine to improve the overall cost-effectiveness of BTM [1]. A comparison between BTM and Integra is provided in Table 2, based on a recent retrospective comparative analysis of animal-derived versus fully synthetic acellular dermal matrices, conducted in 2024 [19].

As this study is mainly a retrospective data collection and analysis, without prospective component, the following limitations were identified. Firstly, there is no control group or comparison group, where other skin substitutes or other reconstruction methods were used. Additionally, no validated scar or quality-of-life scales were used, mainly because of the different age groups including neonates and young children, where the quality of life cannot be accurately assessed. Moreover, the cases were not subcategorised based on the anatomical sites or aetiologies of the defects, showing sample heterogeneity; demonstrating though the versatility of BTM.

Studies comparing BTM results in adults versus paediatric patients are limited. The overall treatment time with BTM may vary and this may be due to the different nature of injuries, the susceptibility to infection and the spectrum of pathogens involved between different age groups [2,6]. Studies that quantify the long-term aesthetic and functional outcomes after BTM application would be a valuable addition to the current literature [3]. Additional suggestions for future research could include prospective multicentre studies, which compare BTM versus other established dermal matrices and evaluate longer follow-up periods, using patient-reported outcome measures (PROMs) in both adult and paediatric populations. A cost-effectiveness analysis would further reinforce the benefits of BTM given the reduced donor-site morbidity.

## 5. Conclusions

The present study demonstrates the versatility of BTM in paediatric reconstructive surgery with a wide range of age, indications and anatomical sites of application with overall satisfying outcomes. The big advantages over other dermal substitutes or skin grafts are that BTM is less prone to infection and offers excellent scarring by preserving the normal skin architecture. Specifically in children, BTM might not require grafting, resulting in spontaneous healing with good scarring. Therefore, BTM should be considered in the reconstructive ladder when discussing defect coverage options in paediatric patients.

## Figures and Tables

**Table 1:** Patient demographics, conditions, anatomical site and indication for BTM application, time interval for grafting and final outcome, including complications.

Patient	Gender	Age	Condition	Anatomical Site/Indication	Time Interval for Grafting	Complications/Outcome
P1	Male	16 y.o.	RDEB	Bilateral hand release	No grafting	Bilateral good thumb abduction, bilateral maintaining webspaces, bilateral digit contractures
P2	Male	12 y.o.	RDEB Previous bilateral hand release with blister skin graft and FTSG (6, 7 and 9 y.o.)	Right hand release followed by left hand release 2 months later	No grafting	Bilateral thumb adduction, bilateral finger refusion, bilateral finger severe contractures. Re-operation planned in the near future
P3	Female	7 y.o.	RDEB	Bilateral hand release	No grafting Left hand re-operation (8 y.o.)	Post 2nd surgery: left thumb abduction, maintaining left webspaces, remaining left finger contractures
P4	Female	14 y.o.	RDEB Previous left hand debridement and separation of 3rd webspace (9 y.o.)	Left hand release followed by right hand release 14 months later	No grafting	Left first webspace and thumb abduction decrease, left finger webspaces contractures, left finger flexion contractures. Left hand planned for re-operation. Right thumb maintaining abduction, maintaining right finger webspaces, right digit contractures
P5	Female	11 y.o.	RDEB Previous correction of pseudosyndactyly of right index and middle finger and grafting (6 y.o.)	Defect after biopsy of suspected scc in right middle fingertip	No grafting	No OT required, good healing process after BTM application, no malignancy identified
P6	Male	8 y.o.	RDEB	Right ear ulcers: external auditory canal, triangular fossa, scapha	No grafting	No satisfying healing, chronic wounds due to RDEB recurrence
P7	Female	10 y.o.	60% flame burn when 6 m.o.	Post-burn contracture release in right ankle	BTM and NPWT 28 days: STSG	Ongoing OT for scar care. Satisfying function of right ankle joint
P8	Male	4 y.o.	93% scald burn when 2 y.o.	Post-burn contracture release in left ankle and then in dorsum of right foot	BTM and NPWT 37 days: STSG in left foot 35 days: STSG in right foot	Ongoing OT, TCA injections, ongoing healing, tight scar in anterior ankles restricting full active ROM, thermoplastic splint
P9	Male	1 y.o.	3% contact burn	Right palm STSG, contractures release and FTSG, FTSG failure, then BTM	23 days: STSG and contracture release and splinting 36 days later: further contracture release	Severe finger contractures, ongoing OT and splinting
P10	Female	1 y.o.	45% scald burn	Bilateral forearms, critical condition, patient unstable for grafting	30 days: BTM removal due to infection	Ongoing healing, scar management
P11	Male	9 y.o.	Dog bite injury	Right thigh, exposed muscle fascia	BTM and NPWT No grafting 4 × 4 cm defect	OT for scar care
P12 Figure 1	Female	12 y.o.	Avulsion/degloving injury	Ulnar side of left ring finger with exposed digital nerve repaired with interposition vein graft, exposed extensor and flexor tendons	23 days: FTSG	Tight scars and stiffness of DIP and PIP joints, ongoing splinting and OT
P13 Figure 2	Female	6 y.o.	Friction burn	Dorsum of right hand over MCP joints with exposed extensor tendons	No grafting Cast application	Wounds fully healed, OT for scar management
P14	Female	9 y.o.	Degloving injury	Left lower leg, exposed muscle	BTM and NPWT 25 days: STSG	Wounds healing well, ongoing scar care
P15 Figure 3	Female	10 y.o.	Road traffic accident/degloving injury	Right occipital bone, exposed calvarium	24 days: STSG 12 days later: re-grafting due to STSG failure	Wound fully healed, ongoing OT and scar care
P16	Male	5 y.o.	Crush injury	Right index finger fasciotomies	No grafting 28 days post-op: haematoma underneath the BTM, treated conservatively	Hypertrophic scar formation, ongoing OT, ulnar deviation at PIP joint due to non-union of middle phalanx fracture
P17	Female	15 y.o.	Friction burn	Left medial elbow scar excision. Previous fasciotomies 32 days ago	BTM and NPWT 35 days: STSG and NPWT	Good healing, ongoing scar care
P18	Female	2 y.o.	Osteomyelitis	Right great toe distal phalanx debridement	No grafting	Wound fully healed, sensation loss due to osteomyelitis
P19	Female	2 d.o.	Unknown infection	Left arm, elbow joint tendons exposed	No grafting	Healing well, complete loss of median, radial and ulnar nerve function/neurolysis, tight left wrist extension, scarring
P20	Female	13 y.o.	Multifocal osteomyelitis, staphylococcal infection	Right medial ankle and leg, joint exposed	BTM and NPWT 40 days: STSG and NPWT	Wound fully healed, right ankle joint stiffness due to osteomyelitis
P21	Female	12 y.o.	Infected pilomatrixoma	Right forearm extensor side, soft tissue defect after debridement	23 days: STSG and NPWT	Wound healing well, OT for scar management
P22 Figure 4	Male	10 m.o.	Streptococcal A septicaemia, septic embolism	Left forearm amputation, exposed bone. Left leg debridement, exposed muscle	BTM and NPWT 24 days: STSG and NPWT	Wound edges overgranulation, good elbow function, small defects in leg wound, scars not affecting ROM
P23	Male	1 y.o.	Meningococcal septicaemia, septic embolism	Left lower limb amputation above knee, left arm amputation above elbow, exposed bone	BTM and NPWT 42 days: further 1 cm debridement of necrotic humerus, STSG and NPWT 51 days later: revision left arm amputation	Completion of OT sessions, rehabilitation centre
P24	Male	5 y.o.	Necrotising fasciitis after chickenpox	Upper back, trapezius muscle exposed	BTM and NPWT 33 days: STSG and NPWT	Wounds healing well, stable scars, no restriction in movements
P25	Female	5 y.o.	Streptococcal A septicaemia, septic embolism	Anterior shin and dorsum of right foot, exposed tendons	28 days: STSG and cast	Good mobilization, ongoing OT, serial casting
P26	Male	10 y.o.	Compartment syndrome, extravasation injury	Right forearm volar aspect, exposed muscles after fasciotomies	BTM and NPWT No grafting	OT, scar management, ongoing splinting
P27	Male	1 m.o.	Neonatal ischemia, NICH	Necrotic ulcer in dorsum of left foot, exposed bone	BTM and NPWT 38 days: STSG and NPWT 12 days later: delayed graft failure	Wound epithelialization, splinting to improve foot position
P28	Male	3 y.o.	Streptococcal A septicaemia, septic embolism	Bilateral thigh amputation, exposed bone. Bilateral forearm debridement, exposed muscle	BTM and NPWT in left stump and left forearm 7 days later: BTM in right stump and right upper arm, BTM replacement in left stump and in left forearm due to haematoma underneath. 25 days: STSG and NPWT	Wounds healing well, hypergranulation in right arm, ongoing OT
P29	Female	1 m.o. Pre-term (28 weeks)	Compartment syndrome, extravasation injury	Right forearm amputation below elbow, exposed bone	No grafting	Wound healing well
P30	Male	9 d.o.	Neonatal compartment syndrome	Right forearm fasciotomy, exposed muscle	No grafting 16 months later: contracture release with serial Z-plasty with neurolysis of median nerve and tendon lengthening of FCR and musculotendinous lengthening of FDS	Casting for 2 years, scar contractures in right wrist, intrinsic function of ulnar nerve
P31	Female	1 m.o. Pre-term (24 weeks)	Neonatal ischemia	Forefoot amputation bilaterally	No grafting	Wounds healing well
P32	Male	5 y.o.	Streptococcal A septicaemia, skin necrosis	Right distal forearm and wrist full-thickness skin necrosis	BTM and NPWT No grafting	Ongoing splinting for right little and ring finger, good finger ROM, full ROM in wrist, OT for scar care

Abbreviations: y.o.: years old, m.o.: months old, d.o.: days old, RDEB: recessive dystrophic epidermolysis bullosa, OT: occupational therapy, FTSG: full-thickness skin graft, STSG: split-thickness skin graft, NPWT: negative pressure wound therapy, ROM: range of motion, MCP: metacarpophalangeal, DIP: distal interphalangeal, PIP: proximal interphalangeal, TCA: triamcinolone acetonide, FCR: flexor carpi radialis, FDS: flexor digitorum superficialis, NICH: non-involuting congenital haemangioma.

**Table 2:** Comparative analysis of BTM versus Integra.

	BTM	Integra
Mean surgery time (*p* = 0.011)	1.632 ± 0.571 h	5.282 ± 5.102 h
Median post-operative hospital stay (*p* = 0.003)	0.95 days	6.60 days
Median post-operative follow-up visits (*p* = 0.012)	5	14
Median duration for complete wound closure (*p* = 0.011)	46.96 days	118.91 days
Infection rate (*p* = 0.022)	0.0%	36.4%
Wound hypertrophy rate (*p* = 0.015)	26.7%	81.8%
Failed wound closure (*p* = 0.003)	6.7%	26.7%
Mean profit per square centimetre (*p* < 0.001)	$10.63	$22.53

## Data Availability

The data presented in this study may be available on request from the corresponding author due to privacy and patient confidentiality.
